# Super-Resolution Live Cell Microscopy of Membrane-Proximal Fluorophores

**DOI:** 10.3390/ijms21197099

**Published:** 2020-09-26

**Authors:** Verena Richter, Peter Lanzerstorfer, Julian Weghuber, Herbert Schneckenburger

**Affiliations:** 1 Institute of Applied Research, Aalen University, 373430 Aalen, Germany; verena.richter@hs-aalen.de; 2 Department of Food Technology and Nutrition, University of Applied Sciences Upper Austria, 4600 Wels, Austria; Peter.Lanzerstorfer@fh-wels.at (P.L.); Julian.Weghuber@fh-wels.at (J.W.); 3 Austrian Competence Center for Feed and Food Quality, Safety and Innovation, 3430 Tulln, Austria

**Keywords:** super-resolution microscopy, fluorescence imaging, SIM, TIRF, glucose transporter, insulin, insulin mimetic drugs

## Abstract

Here, we present a simple and robust experimental setup for the super-resolution live cell microscopy of membrane-proximal fluorophores, which is comparably easy to perform and to implement. The method is based on Structured Illumination Microscopy (SIM) with a switchable spatial light modulator (SLM) and exchangeable objective lenses for epi-illumination and total internal reflection fluorescence (TIRF) microscopy. While, in the case of SIM (upon epi-illumination), cell layers of about 1–2 µm in close proximity to the plasma membrane can be selected by software, layers in the 100 nm range are assessed experimentally by TIRF-SIM. To show the applicability of this approach, both methods are used to measure the translocation of the glucose transporter 4 (GLUT4) from intracellular vesicles to the plasma membrane upon stimulation by insulin or insulin-mimetic compounds, with a lateral resolution of around 100 nm and an axial resolution of around 200 nm. While SIM is an appropriate method to visualize the intracellular localization of GLUT4 fused with a green fluorescent protein, TIRF-SIM permits the quantitative evaluation of its fluorescence in the plasma membrane. These imaging methods are discussed in the context of fluorescence lifetime kinetics, providing additional data for the molecular microenvironment.

## 1. Introduction

Methods of super-resolution microscopy (with a resolution below the Abbe criterion) have been reported for more than 20 years. They include wide-field single-molecule techniques with super-localization (e.g., Stochastic Optical Reconstruction Microscopy, STORM; Photoactivation Localization Microscopy, PALM; or related techniques [1,2,3]), as well as laser scanning microscopy (LSM) with Stimulated Emission Depletion (STED) [4] of the outer regions of an illuminated spot, so that fluorescence is confined to a smaller area than in conventional LSM. MINFLUX technology with the tracking of single molecules in the center of a donut-shaped laser beam [5] combines the advantages of both methods.

Structured Illumination Microscopy (SIM) with a periodical pattern leads to a resolution enhancement of around a factor of two compared to the value given by the Abbe criterion [6,7]. Illumination with three laser beams creates an interference pattern with a high spatial resolution in all three dimensions [8], whereas illumination with two laser beams—e.g., the first diffraction orders of an optical grating or a spatial light modulator (SLM) [9]—generates a two-dimensional interference pattern. For the calculation of a super-resolved image, at least nine structured images with different phases and rotation angles [8,9]—adjusted either by galvano mirrors [10] or by a switchable SLM—are needed. Since the light exposure in SIM is considerably lower than the exposures needed for STED, PALM, or STORM ([11] and references therein), photobleaching and phototoxic damages are widely excluded. This favours SIM for live cell microscopy, in particular if longer exposure times or repeated measurements are required.

Open source software for the calculation of a super-resolution image from nine structured images on the basis of Fourier transformation is available [12]. Since this software suppresses low spatial frequencies from out-of-focus layers in the microscope, SIM images are restricted to thin layers of about 1–2 µm. More selective images of the baso-lateral membrane and adjacent cellular sites can be generated by an evanescent electro-magnetic wave arising upon the Total Internal Reflection Fluorescence (TIRF) of a laser beam. TIRF microscopy has been applied to living cells for almost 40 years with light incidence via a glass or quartz prism whose refractive index exceeds that of the specimen (“prism-type TIRF” [13,14]). Almost 20 years later, “objective-type TIRF” was introduced using a microscope objective lens of very high numerical aperture, so that upon illumination by a small annulus or a light spot close to the edge of the microscope, aperture illumination occurs under total internal reflection, with an evanescent wave penetrating about 100 nm into the sample [15]. This principle has been used, e.g., for standing wave microscopy with a lateral resolution slightly above 100 nm [16,17]. Subsequently, microscopes based on the TIRF-SIM technique were applied to living cells, with up to six interfering laser beams hitting the sample under TIRF conditions [18,19,20]. Here, we present a robust SLM-based SIM technique, which, due to its linear arrangement and to a minimum of optical components in comparison to previous systems (e.g., [18,19,20]), is easy to perform and to implement, and which is finally cost-effective. Our setup represents an improvement of a previously reported system [21], modified for TIRF microscopy as well as for switching between SIM und TIRF-SIM.

At the cell surface, the glucose transporter 4 (GLUT4) facilitates glucose uptake in adipocytes and muscle cells. This process is mainly promoted by insulin via the activation of insulin receptors and amplification of respective downstream signalling. GLUT4-mediated glucose absorption is of particular importance for the regulation of blood glucose levels, and hence GLUT4 plays a major role in the pathophysiology of type 2 diabetes mellitus (T2DM) [22,23,24]. Under conditions of low insulin, GLUT4 is mainly sequestered in small perinuclear vesicles, termed GLUT4 storage compartments (GSCs) [25,26]. On the contrary, stimulation by insulin induces the redistribution of GSCs in the plasma membrane, with subsequent vesicle fusion and GLUT4 membrane insertion. In this regard, insulin mimetic substances, which induce GLUT4 translocation independently of insulin, may represent a novel therapeutic strategy for insulin-resistant T2DM patients. Therefore, recent work of the authors has focused on TIRF microscopy to quantitate GLUT4 translocation [27,28]. SIM and TIRF-SIM, as reported in this paper, will permit a further increase in resolution and improve the visibility of GLUT4 translocation upon stimulation by insulin or insulin-mimetic drugs. Most recently, SIM was used to elucidate the intracellular GLUT4 localization and the role of regulatory protein complexes in GLUT4 exocytosis and pathway synthesis [29,30]. Furthermore, single-molecule approaches such as STERM (sample thinning enhanced resolution microscopy) [31], STED [32], dSTORM [33], PALM [34], and QD (quantum-dot)-based single-particle tracking (SPT) [35] were used to identify the molecular basis of insulin-stimulated GLUT4 translocation and plasma membrane organization, including cluster formation and dissociation. In comparison to the aforementioned single-molecule techniques, SIM and TIRF-SIM do not permit single-molecule resolution, but are appropriate for living cells without fixation, can be used for almost any fluorophore or even intrinsic fluorescence, and can be applied within considerably shorter measuring times (a few seconds or less in comparison with several minutes). Therefore, these techniques appear promising, e.g., for testing insulin-mimetic drugs, and in order to get some additional information on a molecular level, they can be combined with fluorescence spectroscopy or lifetime kinetics. In addition, the light exposure is by several orders of magnitude lower than the light exposures used for PALM, STORM, and STED microscopy. Therefore, the physiological conditions are commonly maintained during and subsequent to experiments. The present paper shows the potential of fluorescence imaging by SIM and TIRF-SIM, gives some preliminary results, and discusses fluorescence lifetime kinetics as an additional method to gain data on the microenvironment of fluorescent molecules.

## 2. Results

### 2.1. Microscopy Setup

**Figure 1:**
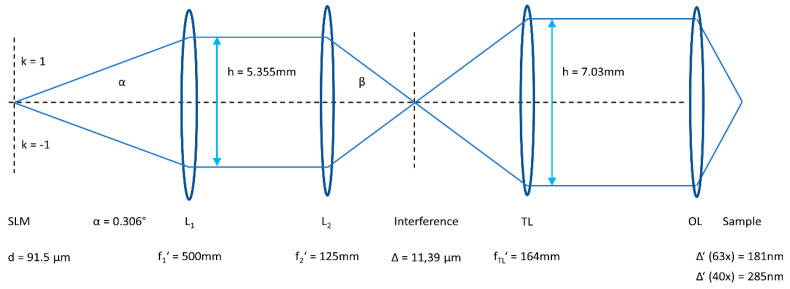
Imaging in SIM and TIRF-SIM (schematic) with a spatial light modulator SLM, telescope lenses L_1_ and L_2_, tube lens TL, and objective lens OL. The illumination of the SLM by the argon ion laser (488 nm) as well as further components (λ/4 plate, pinhole plate, “pizza polarizer”, dichroic mirror, and camera, as reported previously [21]) are omitted. The beam alignment is identical for the SIM and TIRF-SIM apart from the objective lens OL, resulting in different grating constants Δ’ in the plane of the sample (d = grating constant of the SLM; Δ = grating constant of the interference pattern).

The experimental technique is based on a setup for SIM reported previously [21] and is summarized as follows: Illumination occurs by an expanded argon ion laser beam (λ_0_ = 488 nm). Interference patterns resulting from the first diffraction orders of the SLM (ferroelectric LCOS, SXGA-3DM, Forth Dimension Displays, UK) are recorded for three phases (0, 2π/3, 4π/3) and three rotation angles (0°, 60°, and 120°) in order to obtain an optical transfer function (OTF) of rotational symmetry. As depicted in Figure 1, the SLM is imaged by a telescope system consisting of the lenses L_1_ and L_2_, creating an interference pattern which is reduced by the microscope objective lens OL and the tube lens TL. Further components needed for structured illumination are a λ/4 plate, a plate of six pinholes (to select the first diffraction orders of the SLM for 0°, 60°, and 120° orientation) and a so-called “pizza polarizer” to select in each case an azimuthal polarization. All these components could now be maintained for TIRF-SIM. However, the ratio of the focal lengths of the lenses L_2_ and TL had to be adapted so that the distance of opposite interfering beams at the entrance of the objective lens OL used for TIRF (Plan Apochromat 63×/1.46 Oil, Carl Zeiss Jena, Germany) was only slightly smaller than its diameter of 7.63 mm. All the laser beams were thus focused close to the edge of the microscope aperture with an angle of incidence Θ = 66° in the plane of the sample, which exceeds the critical angle of total internal reflection (Θc = 64.7°) and permits a penetration depth of the evanescent electromagnetic field of around 200 nm. Thus, more than 98% of the light in the microscope objective lens was totally reflected. Relevant parameters are given in Figure 1, where additional components (λ/4 plate, pinhole plate, and “pizza polarizer”—all located between the lenses L_1_ and L_2_—as well as a dichroic mirror to deflect the laser wavelength to the sample and record its fluorescence) are omitted. All the parameters are selected such that, by the simple rotation of the microscope lens turret, the TIRF objective lens can be exchanged for a Plan-Neofluar 40×/1.30 oil immersion lens for SIM under epi-illumination. Hereby, the illuminated field changes from 40 (TIRF) to 63 µm, the grating constant in the plane of the sample from 181 to 285 nm, and the resolution (resulting from the Abbe criterion plus the periodicity of the grating) from a theoretical value of 87 to 113 nm. Fluorescence images are recorded by an Orca Flash camera set C11440-10C (Hamamatsu Photonics Deutschland GmbH, Herrsching, Germany), which is synchronized with the SLM. With a pixel size of 3.63 µm, object sizes below 100 nm are well reproduced when using the 40× or the 63× microscope objective lens.

### 2.2. Translocation of GLUT4 to the Plasma Membrane

**Figure 2:**
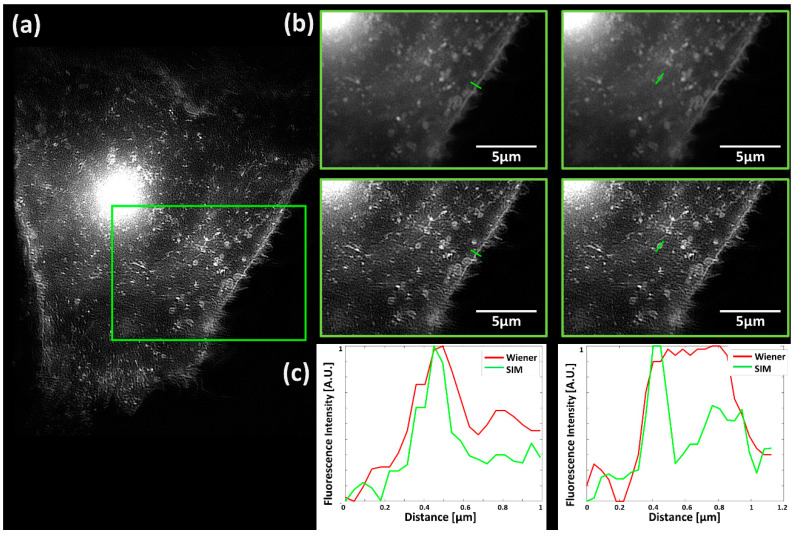
Fluorescence images of a representative CHO-K1-hIR-myc-GLUT4-GFP cell with a focus close to the baso-lateral membrane: (**a**) SIM microscopy; (**b**) comparison of the wide-field image (upper panel, with Wiener filter) and SIM (lower panel) for a selected field; (**c**) line scans over a marked range (as in (**b**) across the plasma membrane (left) and a single vesicle (right) for filtered wide-field microscopy (“Wiener”) and Structured Illumination Microscopy (“SIM”). Excitation wavelength: λ_0_ = 488 nm; detection range: λ_D_ ≥ 515 nm; Plan Neofluar 40×/1.30 oil immersion objective lens.

In order to prove the practicability of the setup described above, we characterized the well-known stimulus-dependent translocation of GLUT4 from intracellular storage compartments to the plasma membrane. For this purpose, insulin as well as insulin-mimetic phytochemicals such as tannic acid and an extract of *Bellis perennis* were used as active agents [27,36]. The potential of SIM in comparison with conventional wide-field microscopy is shown in Figure 2 for a Chinese Hamster Ovary (CHO) cell transfected with GFP-tagged GLUT4 (CHO-K1-hIR-myc-GLUT4-GFP) without further stimulation. GFP-tagged GLUT4 is preferentially located in small vesicles and to some lower extent in the plasma membrane. The improvement of resolution by SIM (around a factor of two) is demonstrated for a selected field and further documented by line scans across the plasma membrane, with a FWHM (full width at half maximum) of about 300 µm (Wiener-filtered image) and less than 180 µm (SIM), as well as a single vesicle. Often, super-resolution microscopy is needed to resolve individual vesicles of 100–200 nm diameter, as shown in Figure 2c.

**Figure 3:**
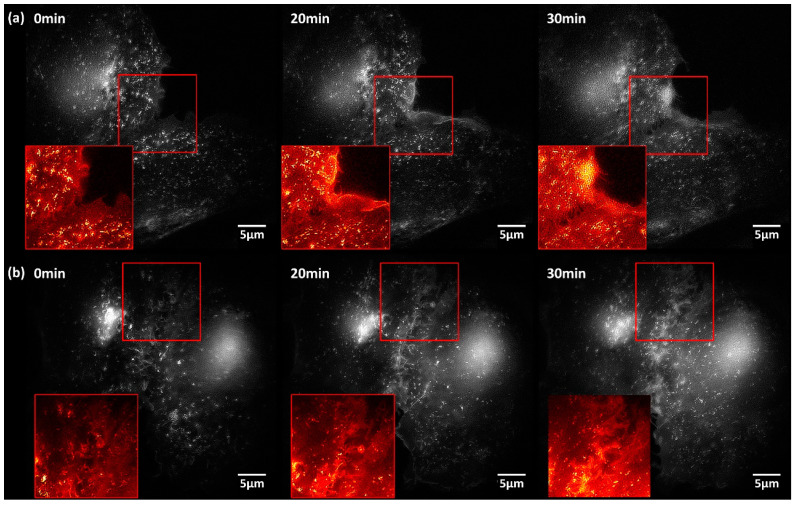
Time course of the fluorescence of CHO-K1-hIR-myc-GLUT4-GFP cells prior to (0 min) and subsequent (20 min, 30 min) to stimulation by (**a**) insulin (1 µM) or (**b**) tannic acid (10 µM); SIM images from a layer close to the baso-lateral membrane. Insets: selected regions of interest; excitation wavelength: λ_0_ = 488 nm; detection range: λ_D_ ≥ 515 nm; Plan Neofluar 40×/1.30 oil immersion objective lens.

Figure 3 shows fluorescence images by SIM of CHO-K1-hIR-myc-GLUT4-GFP cells prior and subsequent to stimulation (20 min, 30 min) by insulin (1 µM, upper part) or tannic acid (10 µM, lower part). In both cases, most of the fluorescence arises again from small vesicles prior to stimulation and is partly re-distributed to larger areas—possibly located within the plasma membrane—after stimulation. In Figure 3, this effect is emphasized in selected regions of interest. The time course of this re-distribution is similar for stimulation by insulin or the insulin mimetic tannic acid.

**Figure 4:**
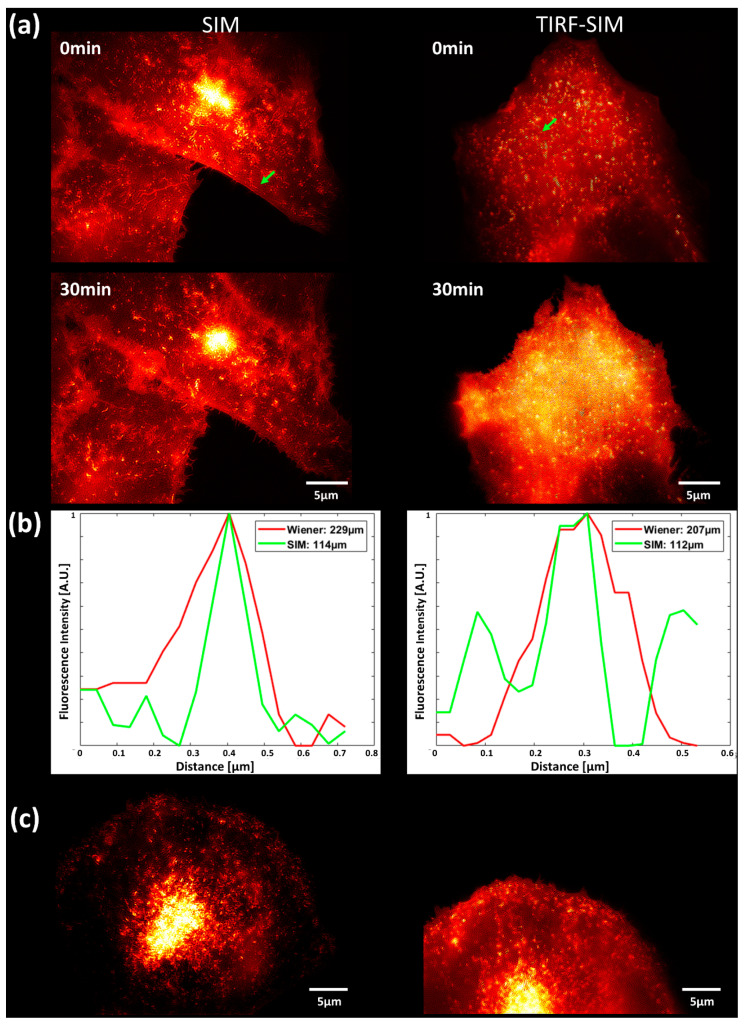
(**a**) Comparison of SIM (left) and TIRF-SIM (right): CHO-K1-hIR-myc-GLUT4-GFP prior to (0 min) and subsequent to stimulation (30 min) with *Bellis perennis* extract (common daisy; 10 mg/L); (**b**) line scans over individual vesicles (marked by an arrow) prior to stimulation for wide-field microscopy (with Wiener filter) and SIM (left) or common TIRF and TIRF-SIM (right); (**c**) comparison of SIM and TIRF-SIM for an identical cell upon change in the objective lens (without stimulation). Excitation wavelength: λ_0_ = 488 nm; detection range: λ_D_ ≥ 515 nm; Plan Neofluar 40×/1.30 oil immersion (left) or Plan Apochromat 63×/1.46 oil immersion objective lens (right).

SIM images of the CHO-K1-hiR-myc-GLUT4-GFP cells showed a similar behaviour after stimulation with *Bellis perennis* extract (common daisy) at a concentration of 10 mg/L: fluorescence arises from small vesicles and—preferentially after simulation—from some larger areas within the plasma membrane (Figure 4a, left). When we measured the plasma membrane almost exclusively by TIRF-SIM (Figure 4a, right), we observed a pronounced increase in fluorescence over the whole surface after stimulation. Furthermore, line scans over individual vesicles prove an increase in resolution between SIM and wide-field microscopy or TIRF-SIM and wide-field TIRF by about a factor of two (from 210–230 nm to about 110 nm; Figure 4b). We recorded SIM and TIRF-SIM images in Figure 4a from different cells, as we did for most of our experiments. The main reasons for this are that object fields are different due to different magnifications, that samples may be slightly shifted when an objective lens is steeped repeatedly in an immersion fluid, and that photobleaching cannot be excluded when several experiments are performed on identical sample areas. However, we show in Figure 4c that SIM and TIRF-SIM images can also be recovered from the same cell.

**Figure 5:**
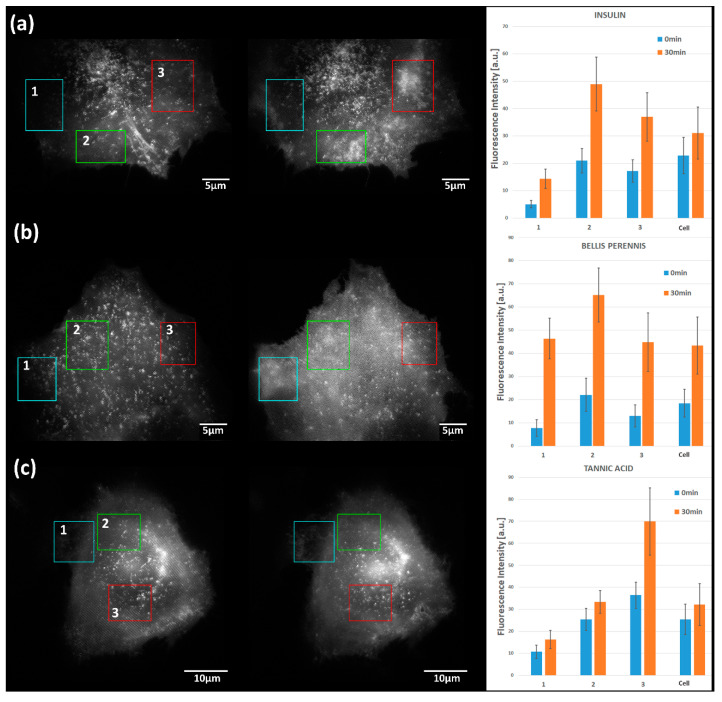
TIRF-SIM images of CHO-K1-hiR-myc-GLUT4-GFP prior to (left) and subsequent to incubation (right) with (**a**) insulin (1 µM), (**b**) *Bellis perennis* extract (10 mg/L), and (**c**) tannic acid (10 µM), including the quantitative evaluation of GLUT4-GFP (means ± standard deviations) for 3 regions of interest as well as for the whole cell. Excitation wavelength: λ_0_ = 488 nm; detection range: λ_D_ ≥ 515 nm; Plan Apochromat 63×/1.46 oil immersion objective lens.

Membrane fluorescence excited by TIRF-SIM was further evaluated in Figure 5 upon stimulation by (a) insulin (1 µM), (b) *Bellis perennis* extract (10 mg/L), or (c) tannic acid (10 µM). In an approach towards the quantitative evaluation of GLUT4-GFP, means ± standard deviations for three selected regions of interest as well as for the whole cell were calculated. For insulin, the increase in fluorescence intensity was by a factor of 1.5–3.0; for the herbal extract *Bellis perennis*, this increase was 2.5–5.0; and for tannic acid it was 1.3–2.0. This shows that with regard to the translocation of GLUT4, the efficiency of the *Bellis perennis* extract was even higher; the efficiency of tannic acid, however, was lower than that of insulin.

## 3. Discussion

The present manuscript describes an application of Structured Illumination Microscopy (SIM). It shows how a custom-built experimental setup for SIM [21] can be extended for TIRF-SIM using a high-aperture microscope objective lens. The focal lengths of the lenses in the illumination beam have to be adapted according to Figure 1, while further components—e.g., λ/4 plate, pinhole, and “pizza polarizer”—can be maintained for the selection of the first diffraction orders of the SLM in three directions with the appropriate (azimuthal) polarizations. This setup is partly related to the equipment described in [19] and allows the use of similar protocols for adjustment. However, beam alignment in a linear configuration and the selection of appropriate polarizations for individual beams (by a λ/4 plate and a simple “pizza polarizer” instead of a combination of polarizer, half wave plate, liquid crystal retarder, and quarter wave plate) is less complex. A further main advantage of the present setup is the switching possibility between TIRF-SIM and SIM (upon epi-illumination) by the simple rotation of the microscope lens turret, since up to the entrance plane of the objective lens the beam alignment is identical for both cases. Thereby, the illuminated object field changes between 40 and 63 µm, but is in both cases sufficiently large to illuminate whole cells. A minor disadvantage may be that there is no fine-tuning of the angle of incidence upon TIRF excitation. In addition, a small fraction of the incident light (≤2%) may illuminate the whole cell. Nevertheless, the setup is well suited for work with living cells growing on microscope cover slips or within micro-well dishes and represents a versatile and low-cost alternative to commercial SIM microscopes (with a principal option for TIRF-SIM), since these systems are commonly stand-alone instruments of high technical potential but limited flexibility.

**Figure 6:**
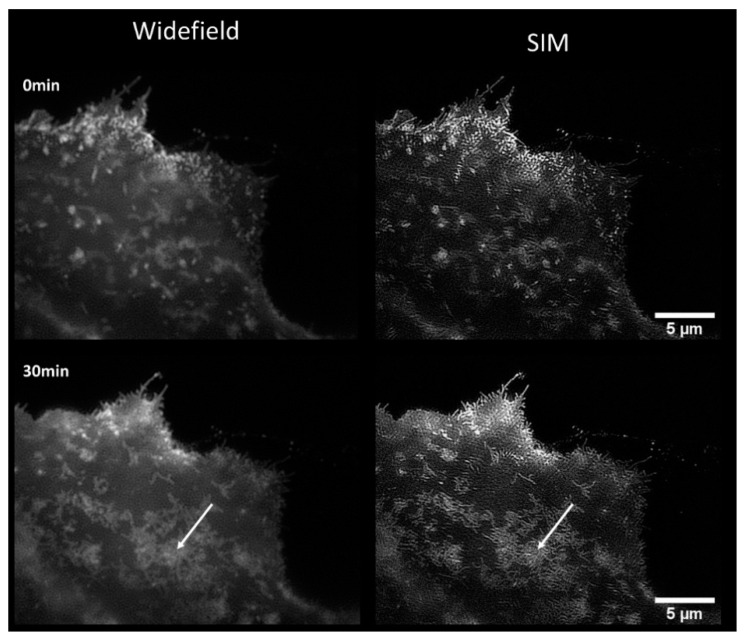
Fluorescence images of MDCK II cells stably expressing GLUT2-mCherry prior and subsequent (30 min) to stimulation with glucose (75 mM); comparison of wide-field microscopy (with Wiener filter) and SIM; arrow indicates membrane area with prominent glucose-stimulated GLUT2 translocation (excitation wavelength: λ_0_ = 488 nm; detection range: λ_D_ ≥ 515 nm; Plan Neofluar 40×/1.30 oil immersion objective lens).

Here, an application of live cell microscopy of the translocation of glucose transporter GLUT4 from intracellular vesicles to the plasma membrane upon stimulation by insulin or insulin mimetic substances is reported. In line with previous studies [27,36], the translocation of GLUT4 induced by bioactive compounds such as tannic acid or the herbal extract of *Bellis perennis* was quantitated based on high-resolution SIM microscopy. To further confirm the applicability of the method, we imaged the translocation of glucose transporter 2 (GLUT2) from intracellular compartments to the plasma membrane. GLUT2 is a facilitative glucose transporter and plays a critical role in glucose homeostasis, as it is mainly expressed in the liver, intestine, kidney, and pancreas [37]. As reported previously [38], the GLUT2 translocation is dependent on the extracellular glucose concentration. In this regard, live cell fluorescence microscopy studies are limited. Therefore, MDCK II cells expressing a GLUT2-mCherry fusion protein were incubated with glucose (75 mM), and, as indicated in Figure 6, SIM identified an increased localization of GLUT2 at membrane proximal areas with a better resolution than that of conventional epi-illumination. Our SIM/TIRF-SIM approach may thus be useful for studying the functional basis of GLUT2 translocation in a live cell context in the future, with minor adjustments to account for the excitation wavelength of mCherry.

In all cases reported above, one can profit from a high lateral resolution of around 100 nm by SIM and a high axial resolution of around 200 nm by TIRF microscopy. The latter one is defined by the penetration depth of the evanescent electromagnetic field, which can be reduced to about 100 nm by increasing the angle of incidence from Θ = 66° to 68–70° if the distance of the opposite interfering laser beams at the entrance of the objective lens OL (Figure 1) is increased from 7.03 to about 7.3 mm. This, however, would require one to replace the focusing lens L_2_ (focal length: f_2′_ = 125 mm) with a lens whose focal length is 122–123 mm, which is not available off-the-shelf. The further improvement of lateral resolution would require, e.g., single-molecule techniques with an increased light exposure and measuring time. In addition, those techniques would need a low number of fluorophores located within a very thin layer (e.g., the plasma membrane of fixed cells) and, in the case of PALM, photo-switchable fluorescent dyes [28]. In contrast, a combination of SIM and TIRF-SIM techniques, as reported in this manuscript, appears appropriate for uses with various fluorophores and concentrations, limited light exposure, a short measuring time, and altogether a limited complexity of the experimental setup.

As documented in our Supplementary Information, these imaging techniques can be combined with fluorescence lifetime measurements in the nanosecond range in order to obtain further data on a molecular level. Fluorescence lifetime τ is very sensitive to the microenvironment of a fluorescent molecule, and in our case a decrease in τ from 2.5–2.8 to 2.3–2.4 ns upon stimulation by insulin or insulin mimetic compounds (see Figure S1) is concomitant with a re-distribution of GLUT4-GFP from intracellular vesicles to the plasma membrane. If, instead of whole cells, the plasma membrane is illuminated selectively by TIRF, a value of around 2.2 ns reflecting the membrane-associated GLUT4-GFP fluorescence remained almost constant after stimulation. Therefore, the fluorescence lifetime may represent a molecular parameter for fluorescence re-distribution from intracellular vesicles to the plasma membrane, as observed in the SIM/TIRF-SIM images.

## 4. Materials and Methods

### 4.1. Reagents

Human insulin and tannic acid were purchased from Sigma-Aldrich (Schnelldorf, Germany). *Bellis perennis* extract was prepared as described previously [36]. For the preparation of stock solutions, substances were dissolved in Krebs Ringer phosphate HEPES buffer (KRPH; 20 mM of HEPES, 1 mM of CaCl_2_, 136 mM of NaCl, 4.7 mM of KCl, 1 mM of MgSO_4_, and 5 mM of KH_2_PO_4_).

### 4.2. Cell Culture

The CHO-K1 cells stably expressing human insulin receptor (hIR) and GLUT4-myc-GFP were a kind gift from Manoj K. Bhat (National Centre for Cell Science, University of Pune, India). The cells were maintained in Ham’s F12 culture medium supplemented with 100 μg/mL of penicillin, 100 μg/mL of streptomycin, 1% G418, and 10% fetal bovine serum (FBS) (all Life Technologies, Carlsbad, CA, USA). For the SIM/TIRF-SIM measurements, 80 cells/mm^2^ were seeded in 8-well/18-well glass bottom chamber slides (ibidi GmbH, Gräfelfing, Germany). Then, 48 or 72 h after seeding, the cells were rinsed with Hank’s balanced salt solution and starved for 3 to 4h in Krebs-Ringer-Phosphate-HEPES (KRPH) buffer. For the (TIRF-)FLIM measurements, 100/150 cells/mm^2^ were seeded on microscope slides. Then, 48 or 72 h after seeding, the cells were rinsed with and starved for 3h in Hank’s balanced salt solution. Insulin (1 µM), tannic acid (10 µM), or *Bellis perennis* extract (common daisy, 10 mg/L) were added on the microscope directly before the measurements. The cells were grown at 37 °C in a humidified atmosphere with 5% CO_2_. For a comparison with glucose transporter 2 (see Discussion), MDCK II cells stably expressing GLUT2-mCherry were used and stimulated with glucose (75 mM).

### 4.3. SIM/TIRF-SIM

The SLM was illuminated by an argon ion laser operated at λ_0_ = 488 nm and with a power of 4 mW (Innova 90, Coherent, Palo Alto, CA, USA). This laser beam was expanded by a telescope to a diameter of 10.5 mm to illuminate a large part of the SLM. Due to pronounced losses by the pinhole plate, the polarizer and microscope optics the incident power on the samples was about 70 µW (epi-illumination) when using the Plan-Neofluar 40×/1.30 oil immersion lens and about 80 µW (TIRF illumination) when using the Plan Apochromat 63×/1.46 oil immersion lens. We checked the angle Θ of light incidence on the sample upon TIRF-SIM after the transmission of a rectangular glass prism, which was optically coupled to the glass bottom where cells were commonly growing, and determined a value Θ = 66°, which exceeded the critical value of total internal reflection Θc = 64.7°. In this case, the penetration depth of the evanescent electromagnetic field was d = λ_0_/[4π (n_G_^2^ sin^2^Θ – n^2^)^0.5^] ≈ 200 nm [13,14], with n_G_ = 1.515 corresponding to the refractive index of the glass bottom, and *n* = 1.37 to that of the cells. Laser light was applied to the samples via a dichroic mirror for 510 nm, and fluorescence was registered at λ ≥ 515 nm using an appropriate long-pass filter.

## Supplementary Materials

Supplementary materials can be found at https://www.mdpi.com/1422-0067/21/19/7099/s1.

## Abbreviations

CHO	Chinese Hamster Ovary
GLUT4	Glucose Transporter 4
GLUT 2	Glucose Transporter 2
PALM	Photoactivation Localization Microscopy
SIM	Structured Illumination Microscopy
SLM	Spatial Light Modulator
STED	Stimulated Emission Depletion Microscopy
STORM	Stochastic Optical Reconstruction Microscopy
TIRF	Total Internal Reflection Fluorescence
