# Supplementary material for: Super-Resolution Live Cell Microscopy of Membrane-Proximal Fluorophores
Source: Int J Mol Sci. 2020 Sep 26;21(19):7099. doi: 10.3390/ijms21197099 (PMC7582769; doi:10.3390/ijms21197099)
Supplement: Supplementary file 1 [file ijms-21-07099-s001.pdf]

*Supplementary Information*

# Super-Resolution Live Cell Microscopy of Membrane-Proximal Fluorophores

**Verena Richter [1], Peter Lanzerstorfer [2,3], Julian Weghuber [2,3] and Herbert Schneckenburger [1,*]**

[1] Institute of Applied Research, Aalen University, 373430 Aalen, Germany; verena.richter@hs-aalen.de
[2] Department of Food Technology and Nutrition, University of Applied Sciences Upper Austria, 4600 Wels, Austria; Peter.Lanzerstorfer@fh-wels.at (P.L.); Julian.Weghuber@fh-wels.at (J.W.)
[3] Austrian Competence Center for Feed and Food Quality, Safety and Innovation, 3430 Tulln, Austria
* Correspondence: herbert.schneckenburger@hs-aalen.de; Tel.: +49-7361-576-3401

## Fluorescence Lifetime Kinetics

The lifetime $\tau$ of an excited molecular state, also termed "fluorescence lifetime", corresponds to the reciprocal of its rates of deactivation $k = k_F + k_{NR} + k_{ET}$ with $k_F$ representing radiative (fluorescent) transitions, $k_{NR}$ non-radiative transitions and $k_{ET}$ energy transfer to adjacent molecules (often assigned as Förster Resonance Energy Transfer, FRET [1]). Therefore, measurement of $\tau$ in the nanosecond time range is often used to describe molecular interactions upon variation of $k_{ET}$ (for a review see e.g., [2–5]). In addition, $\tau$ depends on molecular conformation, intersystem crossing between excited singlet and triplet states or on the microenvironment of relevant molecules, which have an impact on $k_{NR}$. To assess possible changes of the microenvironment we measured the fluorescence lifetime of GLUT4-GFP prior to and subsequent to stimulation with insulin or the insulin-mimetic substances tannic acid or *Bellis perennis* extract and illuminated alternatively whole cells as well as their plasma membranes using an illumination angle of 62° (epi-illumination of whole cells) or 66° (TIRF).

In our Supplementary Figure 1 fluorescence lifetimes of GLUT4-GFP prior to (0 min.) and subsequent to (30 min.) stimulation with insulin, tannic acid or *Bellis perennis* extract are depicted. We determined in each case median values ± MADs (median absolute deviations) from 13–14 object slides each containing 3–5 individual cells illuminated under 62° (whole cell) or 66° (TIRF). Values around 2.2 ns upon TIRF illumination reflecting membrane associated GLUT4-GFP fluorescence remained almost constant after stimulation. In contrast, fluorescence lifetimes of 2.5–2.8 ns within whole cells decreased upon stimulation to 2.3–2.4 ns, thus approaching the values measured within the plasma membrane. This decrease was significant for insulin ($p < 0.05$) and indicated a trend for tannic acid as well as for *Bellis perennis* extract. Therefore, fluorescence lifetime may represent a molecular parameter for fluorescence re-distribution from intracellular vesicles to the plasma membrane as observed in the SIM/TIRF-SIM images.

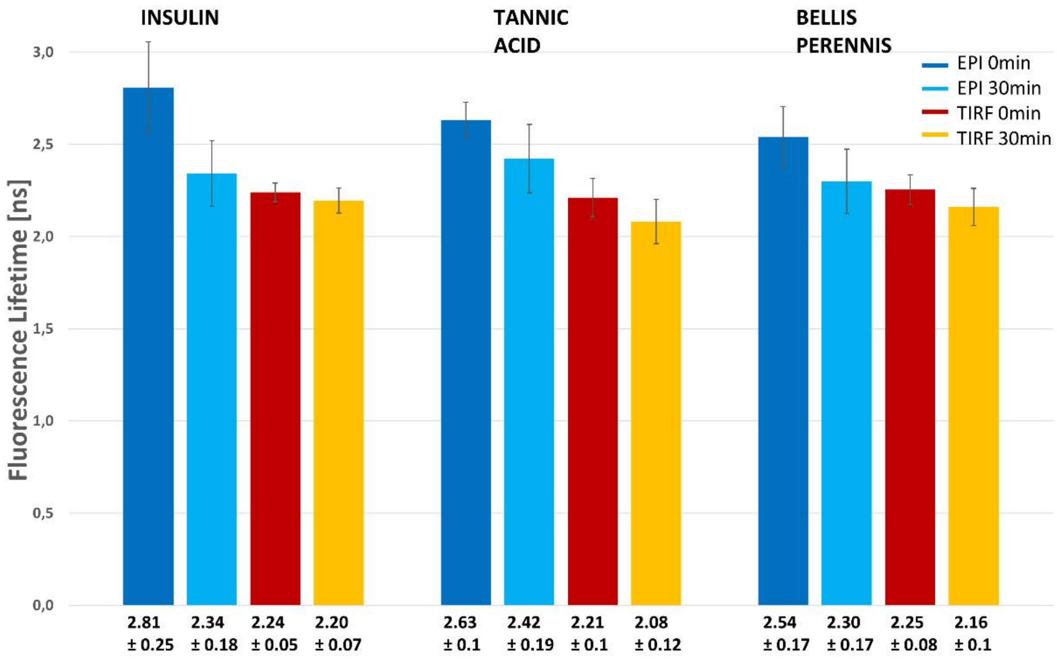


**Figure S1.** Fluorescence lifetime of GLUT4-GFP prior and subsequent to 30 min. stimulation with insulin, tannic acid or *Bellis perennis* extract. Median value ± median absolute deviation (MAD) of 13 (insulin) or 14 (tannic acid, *Bellis perennis* extract) cell specimens in each case upon whole cell illumination (“EPI”) or TIRF.

## Method

Fluorescence decay kinetics was recorded in an upright microscope (Axioplan 1; Carl Zeiss Microscopy GmbH, Jena, Germany) equipped with a custom-made condenser unit with adjustable illumination angles [5] using a super-continuum fiber laser (SuperK with VARIA optical tuning filter, NKT Photonics, Birkeröd, Denmark) for excitation. This laser emitted quasi-continuous pulses of 5 ps duration with a repetition rate of 78 MHz at 450–490 nm. GLUT4-GFP fluorescence from object fields of 100 µm × 150 µm (containing 3–5 individual cells each) on microscope slides was recorded by a 63×/0.90 water immersion lens, a long pass filter for $\lambda \geq 515$ nm and an image intensifying camera system (Picostar HR 12; LaVision, Göttingen, Germany) prior to and subsequent to stimulation with insulin, tannic acid or *Bellis perennis* extract. Time gates of 200 ps were shifted over an axis of 8 ns for measuring fluorescence decay curves, which were evaluated by a mono-exponential fitting program, as further described in [6]. Overlapping background fluorescence was negligible.

## References

1. Förster, T. Zwischenmolekulare Energiewanderung und Fluoreszenz. *Ann. Phys.* **1948**, *437*, 55–75.
2. Clegg, R.M.; Sener, M.; Govindjee. From Foerster resonance energy transfer to coherent resonance energy transfer and back. In *Optical Biopsy VII*; Alfano, R.R., Ed.; SPIE BiOS: San Francisco, California, United States, 2010.
3. Masters, B.R. Paths to Förster’s resonance energy transfer (FRET) theory. *Eur. Phys. J.* **2014**, *H 39*, 87–139.
4. Schneckenburger, H. Förster resonance energy transfer–What can we learn and how can we use it? *Methods Appl. Fluoresc.* **2019**, *8*, 013001, doi:10.1088/2050-6120/ab56e1.
5. Stock, K.; Sailer, R.; Strauss, W.S.L.; Lyttek, M.; Steiner, R.; Schneckenburger, H. Variable-angle total internal reflection fluorescence microscopy (VA-TIRFM): Realization and application of a compact illumination device. *J. Microsc.* **2003**, *211*, 19–29.
6. Schneckenburger, H.; Stock, K.; Lyttek, M.; Strauss, W.S.L.; Sailer, R. Fluorescence lifetime imaging (FLIM) of rhodamine 123 in living cells. *Photochem. Photobiol. Sci*. **2004**, *3*, 127–131.
